# Trimethylamine N-oxide and the reverse cholesterol transport in cardiovascular disease: a cross-sectional study

**DOI:** 10.1038/s41598-020-75633-1

**Published:** 2020-10-29

**Authors:** Laura Bordoni, Joanna J. Samulak, Angelika K. Sawicka, Iwona Pelikant-Malecka, Adrianna Radulska, Lukasz Lewicki, Leszek Kalinowski, Rosita Gabbianelli, Robert A. Olek

**Affiliations:** 1 Unit of Molecular Biology, School of Pharmacy, University of Camerino, Via Gentile III da Varano, 62032 Camerino, MC Italy; 2 Doctoral School, Gdansk University of Physical Education and Sport, 80-336 Gdansk, Poland; 3 Department of Human Physiology, Faculty of Health Sciences, Medical University of Gdansk, 80-210 Gdansk, Poland; 4 Department of Medical Laboratory Diagnostics, Medical University of Gdansk, 80-211 Gdansk, Poland; 5 Biobanking and Biomolecular Resources Research Infrastructure Poland (BBMRI.PL), 80-211 Gdansk, Poland; 6 University Center for Cardiology, 80-211 Gdansk, Poland; 7 Department of Mechanics of Materials and Structures, Gdansk University of Technology, 80-223 Gdansk, Poland; 8 Poznan University of Physical Education, Krolowej Jadwigi 27/39, 61-871 Poznan, Poland

**Keywords:** Risk factors, Biomarkers

## Abstract

The early atherosclerotic lesions develop by the accumulation of arterial foam cells derived mainly from cholesterol-loaded macrophages. Therefore, cholesterol and cholesteryl ester transfer protein (CETP) have been considered as causative in atherosclerosis. Moreover, recent studies indicate the role of trimethylamine N-oxide (TMAO) in development of cardiovascular disease (CVD). The current study aimed to investigate the association between TMAO and *CETP* polymorphisms (rs12720922 and rs247616), previously identified as a genetic determinant of circulating CETP, in a population of coronary artery disease (CAD) patients (n = 394) and control subjects (n = 153). We also considered age, sex, trimethylamine (TMA) levels and glomerular filtration rate (GFR) as other factors that can potentially play a role in this complex picture. We found no association of TMAO with genetically determined CETP in a population of CAD patients and control subjects. Moreover, we noticed no differences between CAD patients and control subjects in plasma TMAO levels. On the contrary, lower levels of TMA in CAD patients respect to controls were observed. Our results indicated a significant correlation between GFR and TMAO, but not TMA. The debate whether TMAO can be a harmful, diagnostic or protective marker in CVD needs to be continued.

## Introduction

Despite significant progress in prevention and treatment strategies of coronary artery disease (CAD), cardiovascular events still constitute the leading cause of mortality and morbidity in the modern world^1^. CAD is characterized by atherosclerosis progressively narrowing the epicardial coronary arteries and impairing myocardial blood flow. The early atherosclerotic lesions develop by the accumulation of arterial foam cells mainly derived from cholesterol-loaded macrophages^2^. Therefore, cholesterol metabolism has been considered as causative in atherosclerosis^3^.

The pathogenesis and potential treatment of the atherosclerotic lesions have been studied using numerous animal models, such as a mouse^4^. However, related to cholesterol metabolism, resistance to atherosclerosis is the major limitation of mouse models^5^. The absence of cholesteryl ester transfer protein (CETP) in mice causes lower plasma cholesterol levels, with high-density lipoprotein (HDL) as the major circulating lipoprotein^6,7^. Thus, genetic modifications, such as low-density lipoprotein (LDL) receptor deficient (LDLR^−/−^) and apolipoprotein E knockout (ApoE^−/−^), have been applied to induce hypercholesterolemia in mice^8–12^. Using a knockout mouse model, trimethylamine N-oxide (TMAO) has been indicated as the key pro-atherogenic compound^13^. High blood TMAO levels activate macrophage influx of cholesterol which leads to foam cell formation and ultimately atherosclerotic lesions^14^. TMAO is produced by the hepatic flavin monooxygenases (FMOs), mainly FMO3, converting trimethylamine (TMA) as a substrate^15,16^. TMA is a waste product of gut microbes, which utilize choline or carnitine as a carbon fuel source. Hence, a link between gut microbes and atherosclerosis has been proposed^13,17,18^. However, in ApoE^-/-^ mice transfected with human CETP, an increase in plasma TMAO was associated with a significantly reduced area of aortic lesions^19^. Nevertheless, recent clinical studies have shown a positive correlation between elevated plasma TMAO and an increased risk for major adverse cardiovascular events defined as death, myocardial infarction, or stroke^20,21^.

According to the current dogma, CETP decreases HDL-cholesterol and increases low-density lipoprotein LDL-cholesterol. Remarkably, genome-wide association studies followed by a Mendelian randomization^22^ have shown that some independent genetic variants (in particular rs12720922 and rs247616), located in the *CETP* gene, largely determine CETP concentration. Per-allele increase in serum CETP was 0.32 µg/mL for rs247616-C and 0.35 µg/mL for rs12720922-A^22^. Moreover, these *CETP* SNPs have been causally associated with lower concentrations of HDL components, while no associations with LDL components have been measured^23^. This demonstrates that rs12720922 and rs247616 are makers able to predict HDL-cholesterol levels, and corroborates the hypothesis that *CETP* can mediate cardiovascular risk by affecting HDL-cholesterol levels. Thus, in accordance with previous evidence on mice model, it can be hypothesized that the different genetic background determining the CETP concentration might modulate the association between TMAO and CVD risk.

Therefore, the aim of the current study was to investigate the association between TMAO and *CETP* polymorphisms (rs12720922 and rs247616), previously identified as genetic determinants of circulating CETP and HDL levels^22,23^, in a population of CAD patients and control subjects with no self-reported medical history of cardiovascular disease (CVD).

## Results

### Descriptive statistics

Among all the 547 enrolled subjects, 358 were male (65.4%), and 189 were female (34.6%). The control group was composed of 153 individuals, while 394 patients suffered from CAD. Descriptive statistics for the analysed variables are displayed in Table 1.

**Table 1:** Characteristics of the study participants.

	Control n = 153	CAD n = 394	p
Age in years	64.3 ± 8.1	66.4 ± 11.7	0.016
Female	64 (41.8)	125 (31.7)	0.028
BMI in kg/m^2^	27.8 ± 4.1	28.8 ± 4.5	0.030
Glomerular filtration rate (GFR)	92.1 ± 31.0	86.6 ± 34.7	0.079
Stable angina	0	196 (49.7)	
Acute coronary syndrome	0	198 (50.3)	
STEMI	0	44 (11.2)	
NSTEMI	0	111 (28.2)	
UA	0	43 (10.9)	
Hypertension	63 (41.2)	301 (76.4)	0.001
Diabetes mellitus	21 (13.7)	118 (29.9)	0.001
Current or past smokers	59 (38.6)	196 (49.7)	0.022

Data are shown as mean ± standard deviation or number (%).

*BMI* body mass index, *GFR* glomerular filtration rate, *STEMI* ST-elevation myocardial infarction, *NSTEMI* non-ST-elevation myocardial infarction, *UA* unstable angina.

### TMAO and TMA in CAD patients and controls

**Figure 1:**
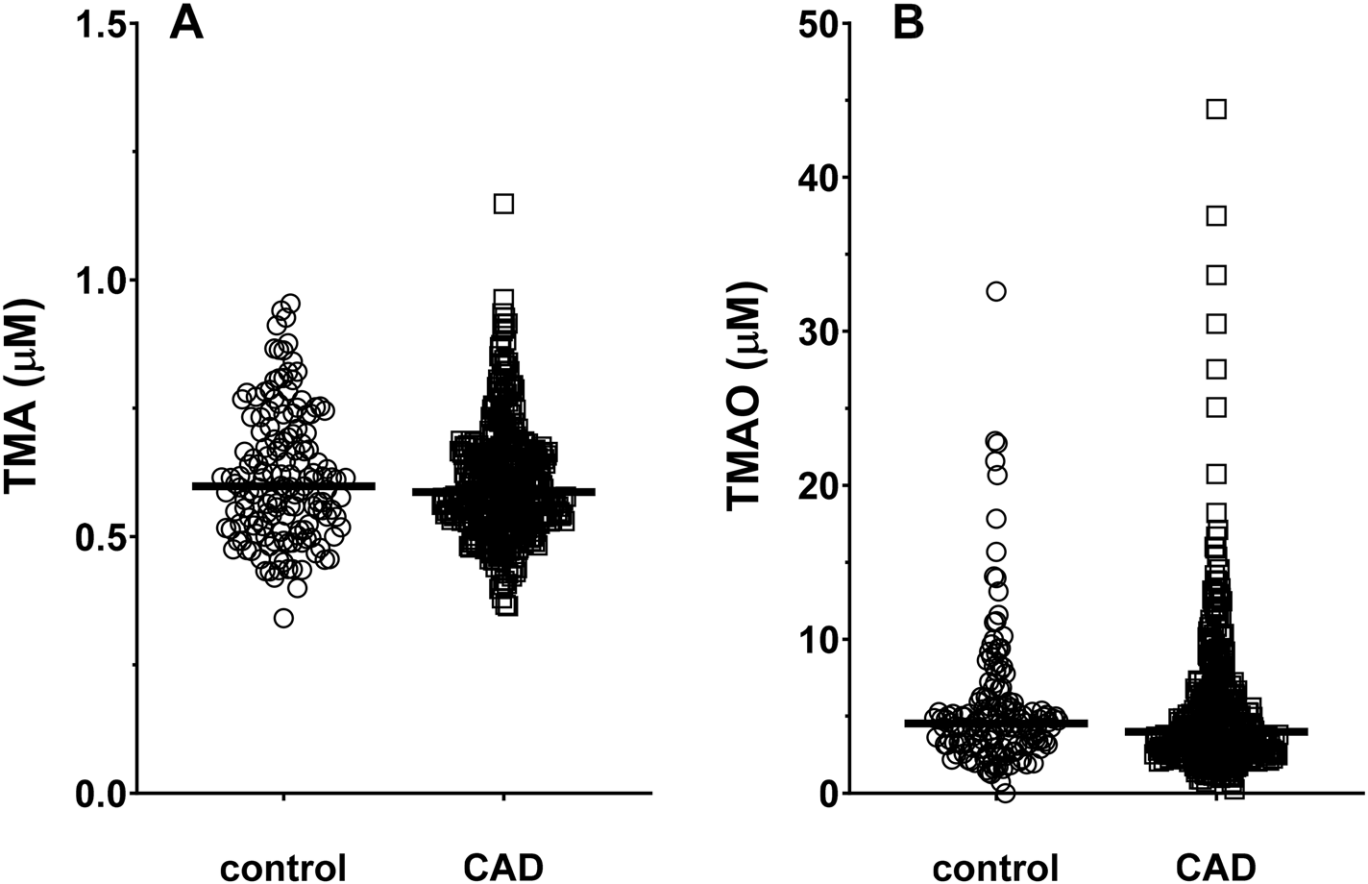
Plasma TMA (**A**) and TMAO (**B**) concentrations in controls (n = 153) and CAD (n= 394) patients. Scatter dot plot with lines as median values.

No differences were noted in row values of plasma TMA between controls 0.62 ± 0.13 μM (mean ± SD) and CAD patients 0.60 ± 0.11 μM (Fig. 1A). However, Generalized Linear Model (GLM) analysis, including adjustments for glomerular filtration rate (GFR), age, body mass index (BMI) and sex, identified a significant difference between the two groups for TMA (expected marginal means ± SD: controls = 0.63 ± 0.01 μM; CAD patients = 0.60 ± 0.01 μM; p = 0.004). TMAO was not significantly different between controls and CAD patients (Fig. 1B), regardless of the row values (p = 0.712) or in the analysis adjusted for the covariates (p = 0.251).

### Genotyping

Genotype and minor allele frequencies of the selected polymorphisms are reported in Table 2. All the polymorphisms were in Hardy–Weinberg Equilibrium (HWE) (p > 0.05) and minor allele frequencies (MAF) at both rs12720922 and rs247616 SNPs were consistent with Northern Europe reference population data (Table 2).

**Table 2:** Genotypic data in the analysed population.

	*CETP* rs247616 n (%)		*CETP* rs12720922 n (%)	
Genotype frequency	CC	238 (43.5)	AA	17 (3.1)
	CT	249 (45.5)	AG	171 (31.3)
	TT	60 (11.0)	GG	359 (65.6)
HWE (P)	0.182		0.384	
MAF current study population	0.337		0.187	
MAF Estonian population (dbSNP)	0.317		0.189	
MAF European population (gnomAD–Genomes)	0.319		0.179	

*MAF* minor allele frequency.

### CETP SNPs are directly associated with HDL-cholesterol levels

Since most of the CAD patients were treated with statins (commonly used as primary or secondary prevention measurement), we relied on a Mendelian randomization-based approach to study the impact of *CETP* and HDL-cholesterol on TMA and TMAO. Despite the potential interference of statins treatment, rs12720922 and rs247616 *CETP* SNPs were significantly associated with HDL-cholesterol levels in the total population (Supplementary Fig. S1 online). Conversely, these polymorphisms were not associated with LDL-cholesterol or total cholesterol levels. This evidence suggests that rs12720922 and rs247616 SNPs can selectively predict HDL-cholesterol even in presence of statin treatment. However, since the risk of unpredictable effects due to the statin treatment cannot be excluded (Supplementary Table S1 online), we confirmed the usage of the Mendelian randomization-based approach for the subsequent analysis and did not consider the raw data on lipid profile.

### CETP SNPs are not directly associated with CAD

Chi-square analysis revealed that genotypes were not differently distributed among controls or CAD patients, thus neither *CETP* rs247616 (p = 0.426) nor rs12720922 (p = 0.488) appear to be directly associated with CVD considering a codominant model. Moreover, no associations were detected using additive models; similarly, no differences in the distribution of alleles between the two classes were detected for any of the analysed SNP (Table 3).

**Table 3:** Differences in genotypic and allelic distributions between controls and CAD patients.

	CAD n (%)	Control n (%)	versus	p
**rs247616**				
CC	178 (45.2)	60 (39.2)	CT + TT	0.208
CT	173 (43.9)	76 (49.7)	CC	0.192
TT	43 (10.9)	17 (11.1)	CC	0.623
CC + CT	351 (89.1)	136 (88.9)	TT	0.947
C	529 (67.1)	196 (64.1)	T	0.334
T	259 (32.9)	110 (35.9)	C	0.334
**rs12720922**				
AA	12 (3.0)	5 (3.3)	AG + GG	0.893
AG	129 (32.8)	42 (27.4)	AA	0.662
GG	253 (64.2)	106 (69.3)	AA	0.992
AA + AG	141 (35.8)	47 (30.7)	GG	0.263
A	153 (19.4)	52 (17.0)	G	0.357
G	635 (80.6)	254 (83.0)	A	0.357

### Effects of different CETP genotypes on TMAO, TMA and TMAO/TMA

**Figure 2:**
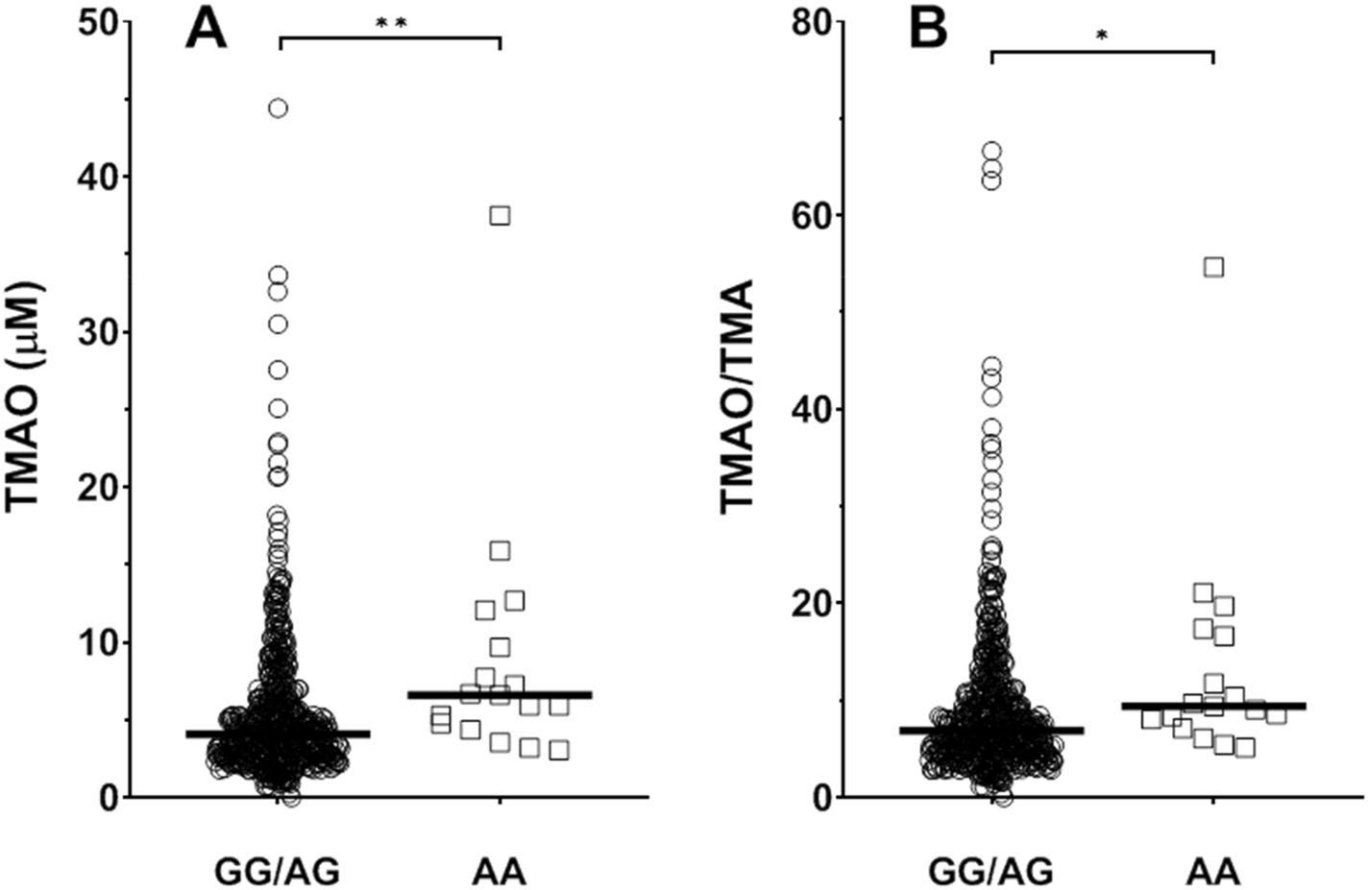
Effect of rs12720922 genotype on plasma TMAO concentrations (**A**) and TMAO/TMA ratio (**B**) in controls and CAD patients. Scatter dot plot with lines as median values. *p < 0.05, **p < 0.01.

*CETP* rs12720922 genotype was associated with TMAO levels (p = 0.008) and TMAO/TMA ratio (p = 0.018) (GLM analysis; sex, age and GFR as covariates; Fig. 2); conversely, it was not linked to TMA levels (p = 0.159). Accordingly, the recessive model resulted in the best fitting, displaying the lowest Akaike’s information criterion (AIC) and Bayesian information criterion (BIC) values both for both rs12720922 (AIC = 3775.1; BIC = 3809.6) and rs247616 (AIC = 3781.2; BIC = 3811.4). Indeed, with respect to rs12720922-AG/GG, rs12720922-AA displayed higher TMAO values (p = 0.004) and higher TMAO/TMA ratio (p = 0.020).

On the contrary, *CETP* rs247616 was not associated with TMAO, TMA, or TMAO/TMA levels (sex, age and GFR as covariates).

### TMAO, TMA in CVD; CETP genetic background association

GLM analysis showed a different association between TMAO or TMAO/TMA levels and health status (controls vs CAD patients) depending on the rs247616 genotype*.* In particular, the rs247616-CC individuals belonging to the control group displayed lower TMAO levels than the carriers of the same genotype in the CAD group. On the other hand, T carriers, that had higher TMAO values in controls, exhibited reduced TMAO levels in the CAD group (P = 0.049) (Supplementary Fig. S2A online). This evidence preliminarily suggested that the increase of TMAO in CAD is typical of those individuals that carry the rs247616*-*CC risk genotype (associated to genetically determined higher CETP and lower HDL levels), but is not generalizable to the entire population. A similar effect was observed for TMAO/TMA ratio, which was different in the control or CAD group depending on the rs247616 genotype (p = 0.046) (Supplementary Fig. S2B online). No significant TMA variations between the control or CAD group were measured neither in dependence on the rs12720922 (p = 0.903) nor the rs247616 (p = 0.569) genotype.

### Haplotype association with CVD, TMAO and TMA

Analysis of haplotypes revealed that it was not possible to demonstrate a cumulative effect of the SNPs from data collected in this study. Indeed, distribution of haplotypes in CAD patients was not different in comparison to controls (p = 0.19) (Table 4).

**Table 4:** Haplotype frequencies estimation (n = 547) in the total population, in controls and CAD groups.

rs247616	rs12720922	Total	Controls	CAD	Cumulative frequency
C	G	0.4829	0.4706	0.4883	0.4829
T	G	0.3297	0.3595	0.3175	0.8126
C	A	0.1798	0.1699	0.183	0.9924
T	A	0.0076	0	0.0112	1

Moreover, there was not a significant association between haplotypes and TMAO (global haplotype association, p = 0.45) nor TMA levels (global haplotype association, p = 0.16) (Table 5).

**Table 5:** Haplotype association with TMAO and TMA in the total population.

	rs247616	rs12720922	Frequency	Difference (95% CI)	p
**(A) Haplotype association with TMAO (n = 547, adjusted by sex + age + BMI + GFR)**					
1	C	G	0.4831	0.00	–
2	T	G	0.3295	0.13 (− 0.51–0.76)	0.69
3	C	A	0.1796	0.31 (− 0.46–1.08)	0.43
rare	*	*	0.0078	− 1.37 (− 4.91–2.17)	0.45
**Global haplotype association p-value** 0.45					
**(B) Haplotype association with TMA (n = 547, adjusted by sex + age + BMI + GFR)**					
1	C	G	0.4829	0.00	–
2	T	G	0.3297	0.02 (0–0.03)	0.039
3	C	A	0.1798	0.02 (0–0.03)	0.059
rare	*	*	0.0076	0.06 (− 0.03–0.16)	0.200
**Global haplotype association p-value** 0.16					

### Other markers

TMAO significantly correlated with GFR (Spearman coefficient = − 0.289; p = 0.001) and age (Spearman coefficient = 0.196; p = 0.000). TMA was associated with GFR (Spearman coefficient = − 0.104; p = 0.015) as well as BMI (Spearman coefficient = − 0.146; p = 0.001).

## Discussion

In this study, we found no association between TMAO levels and genetically determined CETP in a population of CAD patients and control subjects. Moreover, we noticed no differences between CAD patients and control subjects in plasma TMAO levels.

In particular, we investigated two SNPs, rs247616 and rs12720922, as largely determining CETP concentration^22^. An increase in genetically determined serum CETP concentration has been previously associated with decreased total cholesterol concentration and HDL-cholesterol concentration^22^, with *CETP* as an important determinant of HDL-cholesterol, but not affecting LDL-cholesterol concentration and composition^23^. This evidence was essential in the design of this study since direct measurement of HDL- and LDL-cholesterol were not reliable markers in the recruited population, because most of the CAD patients were treated with statins (commonly used as primary or secondary prevention measurement). Results on *CETP* rs247616 genotyping were similar to those previously shown in the Polish population^24^. Despite the comparability in *CETP* rs247616 genotype and the higher number of subjects recruited, we were not able to observe significant differences on the rs247616 genotypes distribution between CAD patients and control groups. Similarly, no significant differences were observed for the rs12720922 genotype, revealing that the risk-alleles were not differently distributed between controls or CAD patients. Thus, we failed to find an association between the HDL-cholesterol increasing genotypes of *CETP* to CVD. It must be noted that genetic mechanisms raising plasma HDL-cholesterol do not decrease the risk of myocardial infarction^25^, and only SNPs affecting LDL-cholesterol levels or both, LDL-cholesterol and HDL-cholesterol levels, influence CVD risk^26^.

Moreover, data collected in the current study did not support the hypothesis that TMAO is directly associated with CVD. We observed similar plasma TMAO levels in patients with confirmed angiographically CAD and control subjects with no medical history of CVD, and plasma TMAO concentration were coherent with values previously measured in the general population^27^. Moreover, no significant pure associations between the *CETP* genotypes and TMAO metabolism has been found. Nevertheless, some aspects of the *CETP* genotype can be mentioned. Firstly, higher TMAO levels have been measured in the rs12720922-AA carriers, which are the subjects with genetically elevated circulating *CETP* and lower HDL-cholesterol levels. On the contrary, rs12720922-G carriers displayed similar levels of TMAO in both groups. However, it must be noticed that the group of s12720922-AA carriers in CAD patients is limited to a very small number of subjects (n = 12), which is 3.0% of examined CAD population. Secondly, preliminary evidence suggested that the association between high TMAO and CAD is peculiar of the rs247616-CC risk genotype (which is associated to higher *CETP* and lower HDL levels), but is not generalizable to the entire population. Thus, the involvement of *CETP* in CAD seems to be more complex than initially hypothesized^24^, and the association between TMAO and CAD might be not as strong as previously suggested^28,29^. In fact, despite previously reported the pro-atherogenic effect of TMAO^13^, recent studies did not observe a positive correlation between plasma TMAO concentrations and atherosclerosis development^30,31^.

Previous evidence suggested an important implication of HDL metabolism in modulating the association between TMAO and atherosclerosis. Firstly, since the production of TMAO is dependent on liver FMO3^15^, genetic variants of *FMO3* have been implicated in a number of diseases^32^ and TMA/FMO3/TMAO has been identified as a key pathway^16,33^. In particular, expression of FMO3 modifications in LDLR^−/−^ mice alters circulating and hepatic lipid levels^16^. Moreover, knockdown of FMO3 reorganizes whole body cholesterol balance by regulation of reverse cholesterol transport^33^. Moreover, in humans, FMO3 is significantly associated with age, gender, and genotype^34^. Indeed, several cofounding factors that mediates the association between TMAO and atherosclerosis has been identified. We have not determined *FMO3* genotype, but differences in TMA/TMAO ratio due to differences in the amount and activity of FMO3 might be present in our population^16, 35^. For this reason, both age and gender were a priori selected as covariates in statistical analyses. Another aspect to consider is that CVD and kidney disease (KD) are closely interrelated^36^ and diminished renal function is strongly associated with morbidity and mortality in heart failure patients^37^. In ApoE^−/−^ mice model of atherosclerosis, the hypercholesterolemia led to early renal dysfunction that can progress into chronic KD^38^. In chronic KD, TMAO elimination from the body fails, causing the elevation of its plasma concentration^39^. Therefore, higher plasma TMAO in humans was suggested as a marker of kidney damage^40^. Since plasma TMAO has been inversely correlated with GFR^41^, some studies suggest that GFR can be a cofounder in this association^42–44^. Moreover, in the end-stage KD patients, not only TMAO but also plasma TMA is elevated^39^. Thus, we also added GFR as a covariate in the analysis investigating the relationship between TMA/TMAO levels and CVD, so we can exclude that GFR could be responsible for the observed results.

Finally, it is worthy of note that chronic, low-dose oral TMAO treatment showed a reduction in diastolic pressure and cardiac fibrosis in spontaneously hypertensive rats^45^. Since TMAO stabilize proteins against various environmental stress factors, including high hydrostatic pressure^46^, TMAO has been suggested as a result rather than a cause of CVD^29^. Thus, not TMAO, but TMA has been suggested as implicated in CVD^47^. In our results, marginally lower levels of TMA in CAD patients respect to controls were observed. Therefore, the microbial origin of TMA is of great interest. Indeed, a major role is played by the microbiome in regulating health and well-being^48^, and dysbiosis of the gut microbiota has been measured in stroke and transient ischemic attack patients whose blood TMAO levels were decreased^49^.

In conclusion, the studied polymorphisms had no direct roles in the development of CVD in the studied Polish population. Moreover, we observed no differences between CAD patients and control subjects in plasma TMAO levels, TMAO which can be affected by intra-individual variation^50^. The debate whether TMAO can be a harmful, diagnostic or protective marker in CVD^28,29,32^ has to be continued.

## Materials and methods

### Participants

CAD patients were consecutively recruited in one hospital with angiographically confirmed CAD or with angina referred to elective or urgent coronary angiography as inclusion criteria. The diagnosis of ST-segment elevation myocardial infarction (STEMI) and non-ST-segment elevation myocardial infarction (NSTEMI) was established according to the Third Universal Definition of Myocardial Infarction, and unstable angina (UA) was diagnosed according to the 2015 ESC guidelines for the management of NSTE-ACS3^51,52^. Control subjects were recruited in the same region amongst the subjects without a self-reported medical history of CVD. The study was approved by the Regional Bioethical Committee (RBC) in Gdansk (KB-27/16 and KB 32–17). All methods were carried out in accordance with relevant guidelines and regulations approved by RBC. Informed consent was obtained from all subjects.

### Samples collection

Venous blood samples were collected in EDTA-containing tubes. The plasma samples were prepared by centrifugation at 1300×*g* for 10 min at 18–25 °C, and were kept frozen at − 80 °C for later TMA and TMAO analysis.

### TMA and TMAO analyses

Plasma TMA and TMAO were determined by the Ultra-Performance Liquid Chromatography (UHPLC) tandem mass spectrometry method, based on the methods described previously^53,54^. UHPLC separation was performer on an XBridge HILIC 3.5 μm (3.0 mm × 50 mm) column on a NEXERA Shimadzu UHPLC system coupled with QT4500 SCIEX. Trimethyl-d_9_-amine HCl (d_9_-TMA) was used as an internal standard. The 3 μM of d_9_-TMA working solution of internal standard (ISWS) was prepared in methanol/acetonitrile (15:85) and 0.1% formic acid (v/v). Calibration samples, QC and plasma samples were prepared by addition 100 μl of cold ISWS to 50 μl of each sample type. All samples were vortexed and kept on ice for 15 min for protein precipitation. Centrifuged samples (14,000 rpm, 4 °C, for 20 min.) were divided into two parts: without dilution which were used for analysis of TMA concentration and diluted (5:95 of ISWS) for analysis of TMAO. The mobile phase was 70% of acetonitrile with 0.1% formic acid (v/v) and 30% of 15 mmol/L ammonium formate with 0.1% formic acid (v/v) at a flow rate of 0.4/min. The mass spectrometer was operated in multiple-reaction monitoring (MRM)-positive electrospray ionization (ESI+). MRM parameters are included in Supplementary Table S2. Mass spectrometer optimized settings were as follows: IonSpray Voltage = 5.5 kV, source temperature = 300 °C, collision gas = 8, curyine gas = 30.0. Calibration curve range was from 0.3 to 30 μM and from 0.1 to 30 μM TMAO and TMA respectively. The limits of quantification (LOQ) were 0.3 μM and 0.1 μM for TMAO and TMA respectively.

### DNA extraction and genotyping

Genomic DNA was extracted from blood using the kit for genomic DNA purification (A&A Biotechnology, Gdynia, Poland) and it was quantified by NanoDrop 2000 (Thermo Scientific, MA, USA) *CETP* rs12720922 and rs247616 were assessed in real-time PCR by TaqMan assays (Thermo Fisher Scientific, MA, USA), according to the manufacturer instructions.

### Statistical analysis

The sample size was calculated through a power analysis performed by G*Power. The effect size of TMAO variation in CAD patients respect to controls was calculated from the study of Tang and colleagues^18^, which has been identified as a high-quality study in the meta-analysis from Qi and colleagues^55^. The calculated effect size is 1.158; thus, to have a power of 0.95, the minimum sample size is 34 subjects (see Supplementary Fig. S3 online).

Power analysis has been performed using G*Power software^56^. The Shapiro–Wilk test was used for the analysis of the normality of data distribution. Spearman correlation, Chi-square test, Kruskal–Wallis test and Generalized Linear Model (GLM) were used to test correlations and significant differences among analysed variables. Hardy–Weinberg equilibrium was calculated for all the Single Nucleotide Polymorphisms (SNPs) analysed. The best fitting model of the association was determined using the Akaike Information Criterion (AIC) and Bayesian Information Criterion (BIC) provided by SNPStats. The model with the lowest AIC and BIC values was considered the best fitting model. Haplotype frequencies estimation and global haplotype association were calculated using SNPstats^57^. If not differently specified, statistical analyses were performed using the SPSS package for Windows, v.20.0 (SPSS Inc, Chicago, IL).

## Supplementary information

Supplementary Information.

## Data Availability

The datasets generated and analysed during the current study are available from the corresponding author on reasonable request.

## Abbreviations

CAD: Coronary artery disease
CVD: Cardiovascular disease
TMA: Trimethylamine
TMAO: Trimethylamine N-oxide
CETP: Cholesteryl ester transfer protein
GFR: Glomerular filtration rate
