# Supplementary material for: Trimethylamine N-oxide and the reverse cholesterol transport in cardiovascular disease: a cross-sectional study
Source: Sci Rep. 2020 Oct 29;10:18675. doi: 10.1038/s41598-020-75633-1 (PMC7596051; doi:10.1038/s41598-020-75633-1)
Supplement: Supplementary file 1 — Supplementary Information. [file 41598_2020_75633_MOESM1_ESM.pdf]

Trimethylamine N-oxide and the reverse cholesterol transport in cardiovascular disease: a cross-sectional study

Laura Bordoni[1*], Joanna J. Samulak[2], Angelika K. Sawicka[3], Iwona Pelikant-Malecka[4], Adrianna Radulska[4], Lukasz Lewicki[5], Leszek Kalinowski[4,6,7], Rosita Gabbianelli[1], Robert A. Olek[8*]

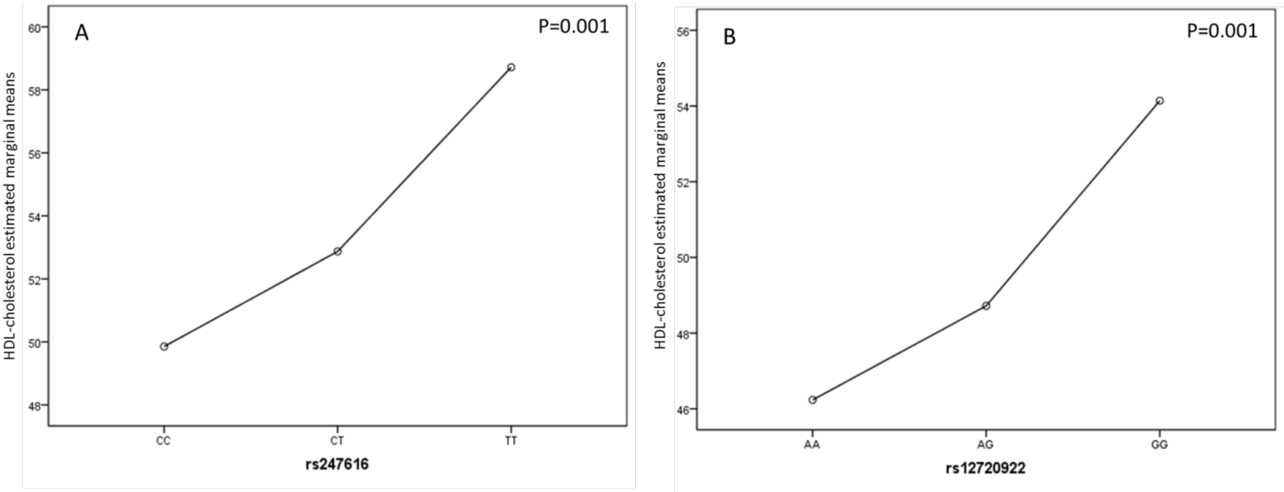

Figure 1S HDL levels dividing the population for the rs247616 (A) and rs12720922 (B) genotypes. rs247616-T carriers show higher HDL levels than rs247616-C carriers (p=0.001); rs12720922-G carriers display higher HDL levels than rs12720922-A carriers (p=0.001). As expected and demonstrated from previous Mendelian randomization studies, these SNPs did not correlate with LDL, TC or TG levels (data not shown), confirming the validity of these genetic variants as selective predictors of HDL.

	**control n=153**	**CAD n=394**	**P**
HDL cholesterol	55.8 ± 16.1	50.7 ± 16.3	< 0.001
LDL cholesterol	127.2 ± 42.6	95.6 ± 41.2	< 0.001
Total cholesterol	208.8 ± 46.1	171.9 ± 46.9	< 0.001
Triglycerides	128.7 ± 74.7	124.8 ± 66.4	0.891

Table S1 Lipid profile in the control (n=153) and CAD (n=394) groups.

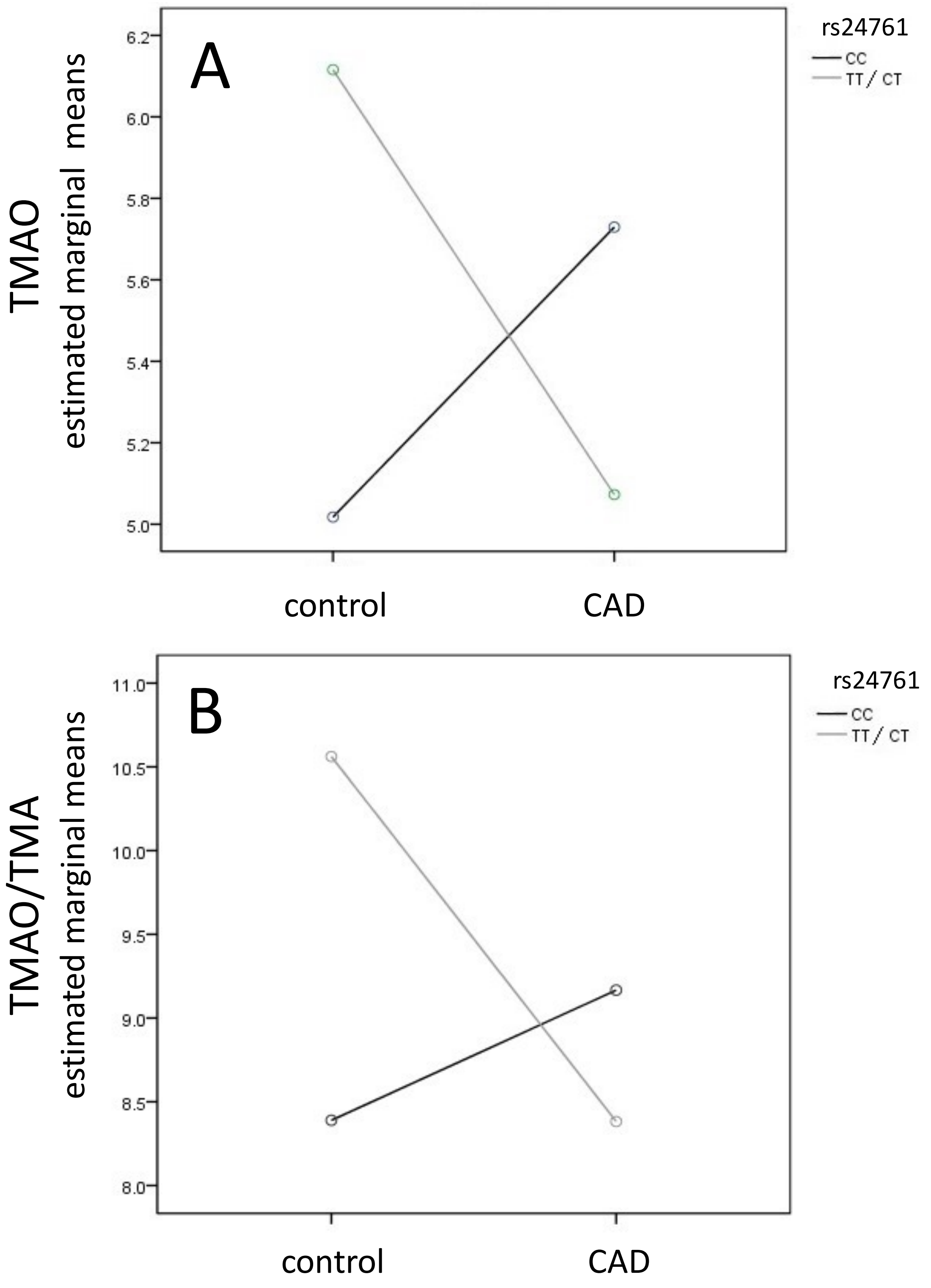

Fig. 2S Effects of rs247616 genotype on TMAO levels (panel A) and TMAO/TMA ratio (panel B) in different health status (controls vs CAD). A) rs247616-CC individuals display lower TMAO levels among the controls, but higher TMAO levels in CVD group. On the other hand T carriers, that have higher TMAO values among controls, exhibit lower levels in the CVD group (P=0.049). B) Similar effects were observed for TMAO/TMA ratio that changes in dependence of the rs247616 genotype (p=0.046)

Compounds	Precursor ion (m/z)	Product ion (m/z)	Collision energy (volts)
TMA	60.1	44.0<br>45.0	27.0<br>19.0
TMAO	76.1	59.0<br>58.0	17.0<br>25.0
d_9-TMA	69.1	49.1<br>51.1	28.0<br>21.0

Table S2 Multiple-Reaction Monitoring parameters of TMA and TMAO determination

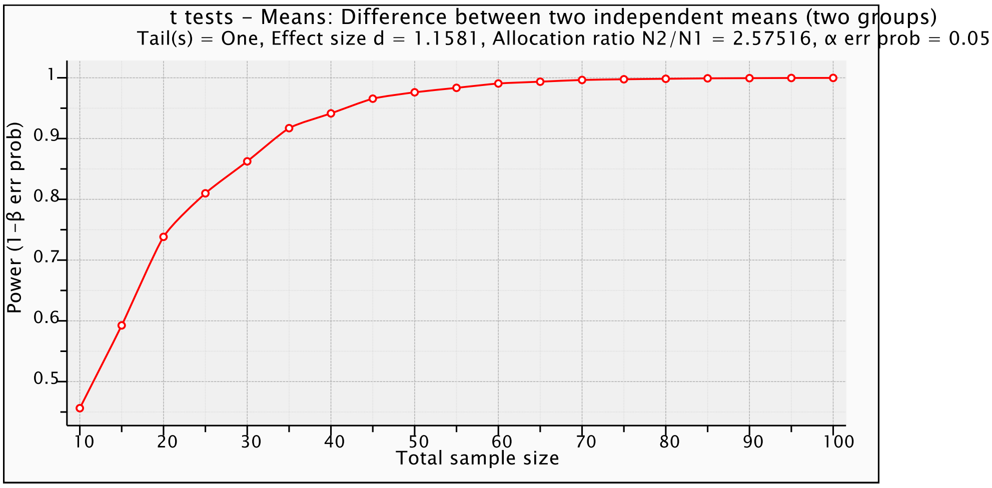

Figure 3S Sample size was calculation through a power analysis performed by G*Power
